# Mechanochemistry assisted asymmetric organocatalysis: A sustainable approach

**DOI:** 10.3762/bjoc.8.240

**Published:** 2012-12-06

**Authors:** Pankaj Chauhan, Swapandeep Singh Chimni

**Affiliations:** 1 U.G.C. Sponsored Centre for Advance Studies in Chemistry, Department of Chemistry Guru Nanak Dev University, Amritsar, 143005, India, Fax: (+)91-183-2258820

**Keywords:** ball-milling, enantioselective synthesis, mechanochemistry, organocatalysis, solvent-free

## Abstract

Ball-milling and pestle and mortar grinding have emerged as powerful methods for the development of environmentally benign chemical transformations. Recently, the use of these mechanochemical techniques in asymmetric organocatalysis has increased. This review highlights the progress in asymmetric organocatalytic reactions assisted by mechanochemical techniques.

## Introduction

Green chemistry involves innovation in chemical research and engineering that encourages the design of processes to minimize the use and production of hazardous materials and also reduce the use of energy [1–4]. These requirements are fulfilled by preventing or minimizing the use of volatile and toxic solvents and reagents, minimizing chemical wastage, development of atom-economical processes and recyclable supported catalyst that are less toxic, biodegradable and can be used at low loading.

To address many of these issues mechanochemical methods such as ball-milling and grinding with pestle and mortar have emerged as powerful techniques [5–12]. The mechanical energy generated by grinding two solids or one solid and one liquid substance results in the formation of new surfaces and cracks by breaking the order of the crystalline structure, and this results in the formation of products [8].

Grinding and ball-milling are widely applied to pulverize minerals into fine particles, in the preparation and modification of inorganic solids. Recently, their use in synthetic organic chemistry has increased considerably, due to the need for development of sustainable methodologies, and has been widely used in solvent-free non-asymmetric transformations.

On the other hand, demands for the development of stereoselective synthesis of organic molecules have noticeably amplified in recent times. In this regard, catalytic asymmetric synthesis involving the use of chiral organocatalysts has emerged as a powerful tool from the infancy to the maturity of asymmetric organocatalysis [13–26]. The use of organocatalysts for catalysing asymmetric reactions may allow several advantages, such as lower toxicity compared to metal analogues, robustness, no requirement of an inert atmosphere, provision of high stereoselectivity, and the ability to be used for the synthesis of opposite enantiomers by using enantiomeric catalysts. Organocatalyts also provide an insight into biological catalytic processes, as a number of these catalysts work by the phenomenon of enzyme mimicry. These advantages of chiral organocatalysts also meet many of the requirements of green chemistry [27].

Recently developed, organocatalytic asymmetric transformations assisted by mechanochemical techniques proved to be an excellent alternative to atom-economical stereoselective transformations under solvent-free reaction conditions. This review gives an overview of the solvent-free asymmetric organocatalytic transformation assisted by mechanochemical techniques, viz. ball-milling and grinding with pestle and mortar.

## Review

### Aldol reaction

**Scheme 1:**
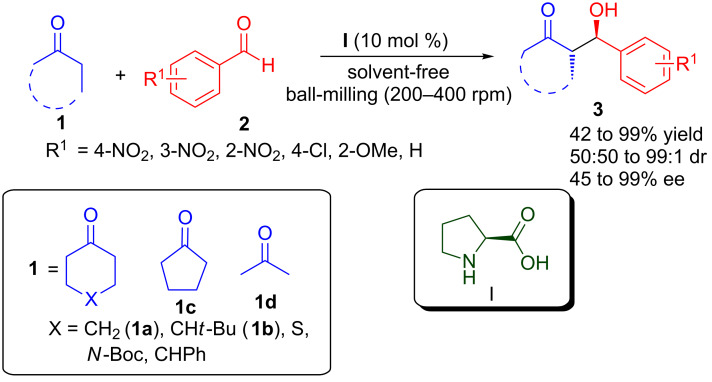
Proline-catalysed aldol reaction in a ball-mill.

Since the origin of organocatalysis, the asymmetric aldol reaction has been one of the most intensely studied reactions, providing an easy access to chiral β-hydroxycarbonyl compounds, which are important building blocks for various bioactive molecules [28]. Among different organocatalysts used for asymmetric aldol reactions, proline and its derivatives emerged as powerful catalysts for the enamine activation of donor aldehyde or ketone. Bolm’s group reported a solvent-free asymmetric organocatalytic aldol reaction under ball-milling conditions using L-proline (**I**) as catalyst (Scheme 1) [29–30]. Various five and six-membered cyclic ketones **1** were reacted with aromatic aldehydes **2** to provide *anti*-aldol products **3** in good to excellent yield (42–99%) and poor to high diastereoselectivity (50:50 to 99:1 dr) and moderate to excellent enantioselectivity (45 to >99% ee). Cyclohexanone derivatives resulted in high stereoselectivity of *anti-*aldol products; however, in the case of cyclopentanone poor diastereoselectivity was observed. The advantages of ball-milling technique over traditional stirring in the proline-catalysed aldol reaction can be highlighted as follows: (1) faster reaction rate, which leads to high yield of aldol products with excellent stereoselectivity; (2) clean reactions providing predominantly crystalline solids, which can be easily isolated; and (3) uneconomical and impractical use of a large excess of ketone could be avoided, as almost equimolar amounts of the starting materials were employed. Further, the entirety of the starting materials was consumed, which facilitated the isolation of the product from the reaction mixture.

**Scheme 2:**
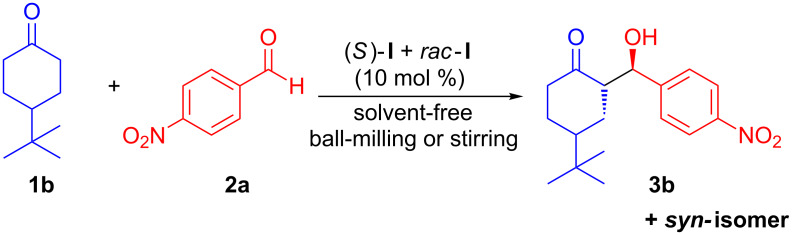
Proline-catalysed aldol reaction between solid substrates (**1b** and **2a**).

The phase behaviour study of the proline-catalysed aldol reactions between solid substrates (**1b** and **2a**) under solvent-free ball-milling reveals a significant nonlinear relationship between the enantioselectivity of the proline and that of the aldol product **3b** (Scheme 2) [31]. This nonlinear behaviour was thought to originate from the ternary phase behaviour of scalemic proline. Neither a phase change nor the product formation was observed upon stirring of mixtures of solids **1b**, **2a** and proline at room temperature. However, heating the reaction mixture to 42 °C resulted in the completion of reaction in 72 hours, and a linear relationship between the enantioselectivity of proline and that of the aldol product was observed.

Subsequently, Bolm and co-workers also reported an enantio-enrichment of a scalemic aldol product **3b** by iterative *retro*-aldol/aldol reactions of *anti*-aldol product in the presence of an achiral or racemic catalyst, i.e., pyrrolidine, by magnetically stirring a solution of the *anti*-aldol product (800 rpm) in DMSO, with ZrO_2_ beads as the grinding medium [32]. The enantioselectivity of the aldol product increases from an initial 70% ee to 92% ee after one day. Subsequently, after two and eleven days, a slow enantio-enrichment (95% ee and 96% ee, respectively) was observed.

**Scheme 3:**
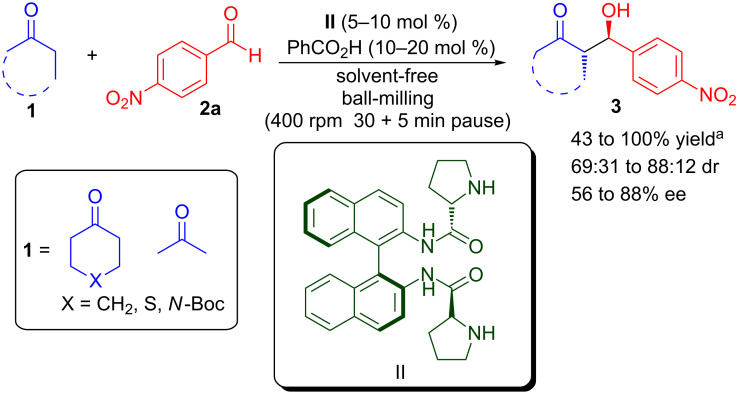
(*S*)-Binam-L-prolinamide catalysed asymmetric aldol reaction by using a ball-mill. ^a^Conversion.

The ball-milling approach was applied to the (*S*)-BINAM-L-prolinamide (**II**) catalysed direct aldol reaction between ketones and aldehydes under solvent-free conditions by Najera and co-workers (Scheme 3) [33–34]. Using 5–10 mol % of **II** and 10–20 mol % of benzoic acid as additive, the aldol reaction of ketones **1** and 4-nitrobenzaldehyde (**2a**) proceeded well and the corresponding aldol products **3** were obtained in moderate to excellent yield (43–100%), low to good diastereoselectivity (69:31 to 88:12 dr), and moderate to good enantioselectivity (56–88% ee). However, no real advantage of ball-milling over traditional stirring in terms of reaction rate, product yield, and stereoselectivity was observed.

**Scheme 4:**
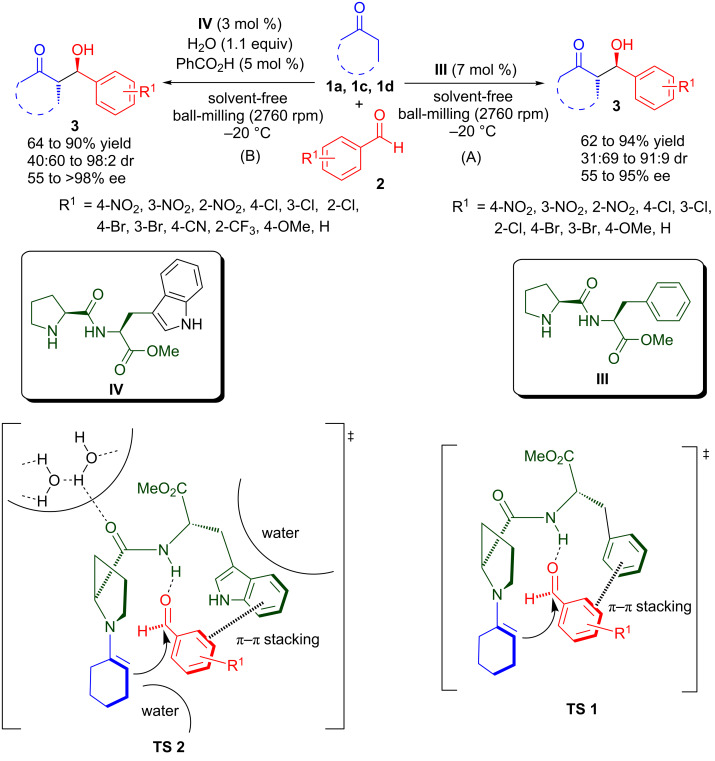
Asymmetric aldol reaction assisted by ball-milling catalysed by dipeptides (A) with **III** and (B) with **IV**.

In 2011, Hernández and Juaristi utilized the catalytic potential of α,α-dipeptide, i.e., methyl ester of (*S*)-proline-(*S*)-phenylalanine (**III**) for organocatalytic asymmetric aldol reaction of cyclohexanone, cyclopentanone and acetone with various aromatic aldehydes **2** under solvent-free reaction conditions with the high-speed ball-milling (HSBM) technique (Scheme 4) [35]. Using 7 mol % of **III** the *anti*-aldol products (in the case of cyclohexanone) and *syn*-aldol product (in the case of cyclopentanone) were obtained in good to high yield (62–94%), moderate to high diastereoselectivity (31:69 to 91:9 dr, *anti*/*syn*) and moderate to high enantioselectivity (55–95% ee). Under HSBM conditions, **III** also catalyses the aldol reaction of acetone with **2a** to provide the corresponding aldol product in 82% yield and 69% ee. In the previous studies by Szöllösi’s group on the dipeptide **III** catalysed asymmetric aldol reactions between acetone and 2-ethylbutanal in the presence of an excess of acetone (68:1) under traditional stirring affords (*R*)-β-hydroxyketone in moderate yield and 86% ee after 24 hours [36]. The proposed transition state (**TS 1**) involves enamine formation between the pyrrolidine unit of the catalyst with the ketone and synergic activation of the aldehyde by hydrogen bonding between carbonyl oxygen and amidic NH of the catalyst. Higher stereoselectivity under solvent-free reaction conditions in comparison to reactions carried out in solvent could be attributed to lower molecular motion due to increased hydrogen bonding between aldehyde and amidic NH, and more effective π–π-stacking interaction between the phenyl ring of the catalyst and aldehyde.

The methyl ester of (*S*)-proline-(*S*)-tryptophan (**IV**) was shown to be an efficient organocatalyst for asymmetric aldol reactions of ketones with aromatic aldehydes in the presence of water by using HSBM (Scheme 4) [37]. The corresponding aldol products **3** were obtained in good yield (64–90%), low to high diastereoselectivity (40:60 to 98:2 dr, *anti*/*syn*) and moderate to excellent enantioselectivity (55 to >98% ee) by using only 3 mol % of **IV**. A similar transition state (**TS 2**) to that of **III** could be proposed, which involves the formation of enamine and simultaneous hydrogen bonding activation of the aldehyde by amidic NH of the catalyst. The large surface area of the lipophilic residue of the tryptophan, reinforced by the hydrophobic environment created by the addition of water, appears to be responsible for the improvement in diastereoselectivity. These factors also enhance the π–π stacking between the catalyst and aldehyde to form a more rigid transition state, which induces higher stereoselectivity. In addition, the acidity of the amidic N–H bond is increased by the formation of a hydrogen bond between water molecules and amidic carbonyl, thus providing a stronger hydrogen-bonding interaction with the aldehyde.

**Scheme 5:**
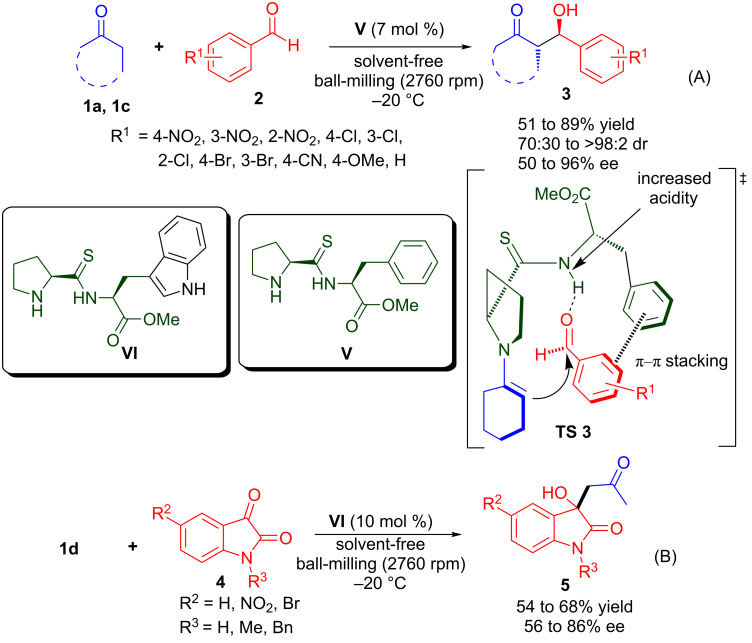
Thiodipeptide-catalysed asymmetric aldol reaction of (A) ketones with aldehydes and (B) acetone with isatin derivatives.

Recently, Juaristi and co-workers reported a highly efficient asymmetric aldol reaction of cyclic ketones with various aromatic aldehydes catalysed by a new series of (*S*)-proline containing thiodipeptides under solvent-free HSBM conditions (Scheme 5) [38]. The thiodipeptide **V** catalyses the stereoselective formation of aldol products in 51–89% yield and moderate to high stereoselectivity (70:30 to >98:2 dr, *anti*/*syn* and 50–96% ee). It was proposed that the thiodipeptide catalyst possesses increased acidity of the thioamidic N–H compared to the amide analogue, which results in a stronger hydrogen-bonding interaction with the aldehyde carbonyl in the transition state (**TS 3**). Under ball-milling conditions, thiodipeptide **VI** catalyses the enantioselective aldol reaction of acetone (**1d**) with isatin derivatives **4** to provide 3-substituted-3-hydroxyoxindole derivatives **5** in moderate yield (54–68%) and moderate to good enantioselectivity (56–86% ee). The reactions carried out under HSBM afford (*S*)-3-hydroxyindole with higher enantioselectivity, relative to the reaction performed under traditional stirring in solvent.

### Michael reaction

**Scheme 6:**
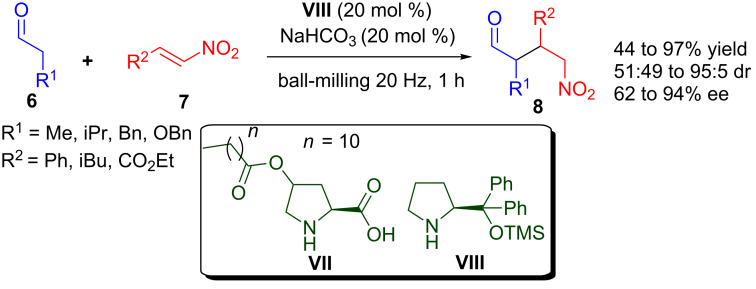
Enantioselective Michael reaction of aldehydes with nitroalkenes catalysed by pyrrolidine-derived organocatalysts.

The asymmetric organocatalytic Michael addition to various unsaturated acceptors is one of the most highly studied and important reactions for the synthesis of valuable chiral molecules [39–44]. Recently, Sebesta and co-workers compared the enantioselective organocatalytic Michael addition of aldehydes to nitroalkenes in aqueous solution with the reaction performed under solvent-free ball-milling conditions catalysed by pyrrolidine-derived organocatalysts (Scheme 6) [45]. Both the procedures provided the product in good yield, poor to high diastereoselectivity, and moderate to high enantioselectivity. Two different organocatalysts were identified for aqueous and solvent-free ball-milling conditions. *O-*Lauroyl*-trans*-4-hydroxyproline (**VII**) was identified as the best catalyst in aqueous media, whilst α,α-diphenylprolinol trimethylsilyl ether (**VIII**) turned out to be the best catalyst under ball-milling conditions. Michael reaction of aliphatic aldehydes **6** with nitroalkenes **7** proceeded rapidly in the presence of 20 mol % of **VIII** under solvent-free ball-milling conditions to provide the desired Michael adducts **8** in 44–97 % yield, 51:49 to 95:5 dr and 62–94% ee. It was also observed that the combination of an organocatalyst with solvent-free ball-milling was more efficient than with conventional stirring, because when the reaction was carried out by conventional stirring, the product yield and stereoselectivity deteriorated significantly.

**Scheme 7:**
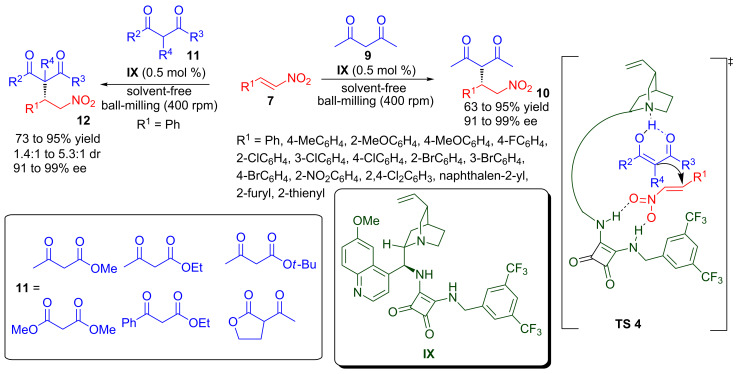
Chiral squaramide catalysed asymmetric Michael reaction assisted by ball-milling.

Xu and co-workers reported a highly enantioselective organocatalytic Michael reaction of 1,2-carbonyl compounds to nitro-olefins under solvent-free conditions by using a planetary ball mill (Scheme 7) [46]. Cinchona-derived chiral squaramide **IX**, at low catalyst loading of 0.5 mol %, efficiently catalyses the solvent-free Michael reaction of acetylacetone (**9**) with various substituted nitroalkenes **7** in a short reaction time (5–30 minutes) under ball-milling (400 rpm) to provide an easy access to Michael adducts **10** in good to high yield (63–95%) and excellent enantioselectivity (91–99% ee). The chiral squaramide **IX** also catalyses the Michael reaction of β-ketoesters **11** with nitrostyrene to provide Michael adducts **12** in 73–95% yield, 1.4:1 to 5.3:1 dr and 91–99% ee. The solvent-free ball-milling of dimethyl malonate with nitrostyrene in the presence of **IX** provides the corresponding Michael adduct in 80% yield and 91% ee. However, chiral squaramide catalysed Michael addition of dicarbonyl compounds to nitroalkenes in dichloromethane by traditional stirring proceeds at a slower reaction rate as compared to ball-milling [47]. The transition state (**TS 4**) for this transformation involves a hydrogen-bonded ternary complex of catalyst and substrates in which two NH groups of the squaramide moiety activate the nitroalkene and the quinuclidine nitrogen activates and orients the dicarbonyl compounds to provide the Michael adduct in high enantioselectivity.

**Scheme 8:**
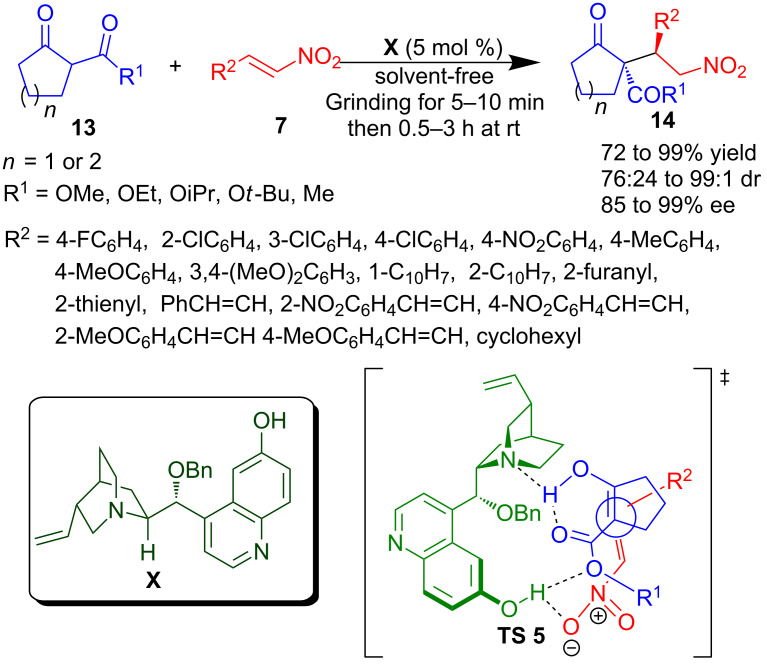
Asymmetric organocatalytic Michael reaction assisted by pestle and mortar grinding.

Application of grinding with pestle and mortar for highly stereoselective Michael addition of trisubstituted β-ketoesters to nitroalkene derivatives was reported by Chimni’s group (Scheme 8) [48]. Grinding an equimolar quantity of six/five-membered cyclic β-ketoesters **13** and various nitroalkenes **7**, including nitrodienes, in the presence of 5 mol % of cupreine-derived organocatalyst **X** provided Michael adducts **14** in good to high yield (72–99%) and good to excellent stereoselectivity (85–99% ee and 76:24 to 99:1 dr). It was observed that the reaction proceeds much faster under grinding conditions, when compared with the reaction carried out under traditional stirring in toluene as solvent or under neat conditions. This was attributed to the fact that grinding facilitates the proper mixing of the catalyst and substrates and also provides additional mechanical pressure. The proposed transition state (**TS 5**) involves a hydrogen-bonded ternary complex of substrates and catalyst, in which the aromatic hydroxy group activates the nitro group of nitroalkene while the tertiary amine of the catalyst activates and orients the β-ketoester.

### Morita–Baylis–Hillman (MBH) reaction

**Scheme 9:**
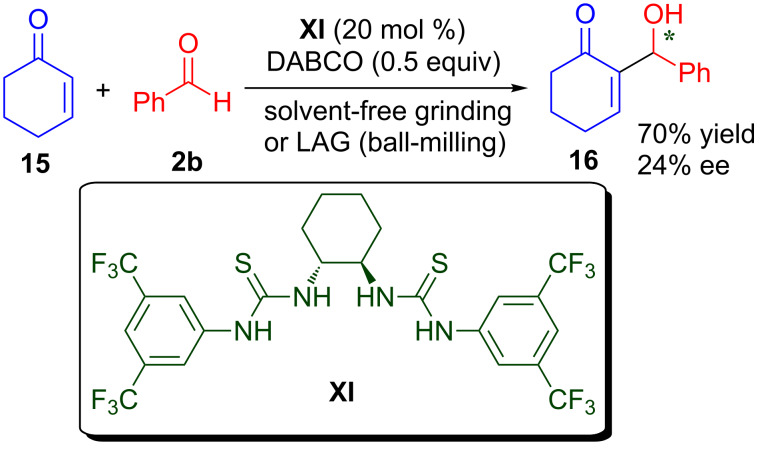
*C*-2 symmetric thiourea catalysed enantioselective MBH reaction.

The Morita–Baylis–Hillman (MBH) reaction provides a very useful and interesting method for the synthesis of β-hydroxycarbonyl compounds with an α-alkylidene group [49–53]. Mechanochemical methods of neat grinding and liquid-assisted grinding (LAG) have been applied to the synthesis of mono- and bis(thiourea)s in quantitative yield by using the click coupling of aromatic or aliphatic diamines with aromatic isothiocyanates (Scheme 9) [54]. The mechanochemically prepared chiral molecules were applied as organocatalysts in an enantioselective MBH reaction and as cyanide ion sensors in organic solvents. Chiral bis-thiourea **XI** catalyses the MBH reaction of benzaldehyde (**2b**) and 2-cyclohexen-1-one (**15**) to provide MBH adduct **16** in 70% yield and 24% ee under neat grinding and LAG.

### Anhydride ring opening

**Scheme 10:**
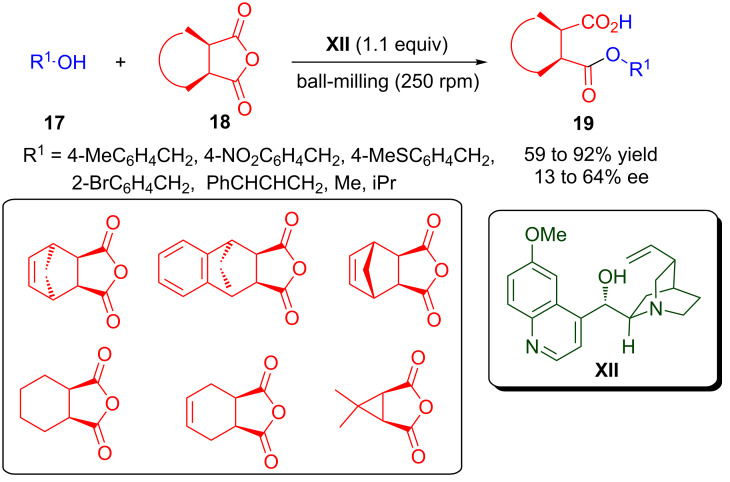
Quinine-catalysed ring opening of *meso*-anhydride by ball-milling.

The stereoselective *meso*-anhydride ring opening represents an important approach for providing multiple stereogenic centres in the target molecule [55–57]. In this feat the Cinchona alkaloids have emerged as powerful organocatalysts. A ball-milling-assisted highly efficient asymmetric ring opening of *meso*-anhydride with alcohols catalysed by quinine (**XII**) was developed by Bolm’s group (Scheme 10) [58]. A variety of *meso*-anhydrides **18** were opened with a variety of alcohols **17** under solvent-free conditions to provide optically active dicarboxylic acid monoesters **19** in good to high yield (59–92%) and low to moderate enantioselectivity (13–64% ee). This methodology have several advantages such as solvent-free reaction conditions, a simple work-up procedure, no column chromatography, use of an almost equimolar substrate, wide substrate scope, and the fact that even two solid substrates react efficiently.

### Alkylation of imine

**Scheme 11:**
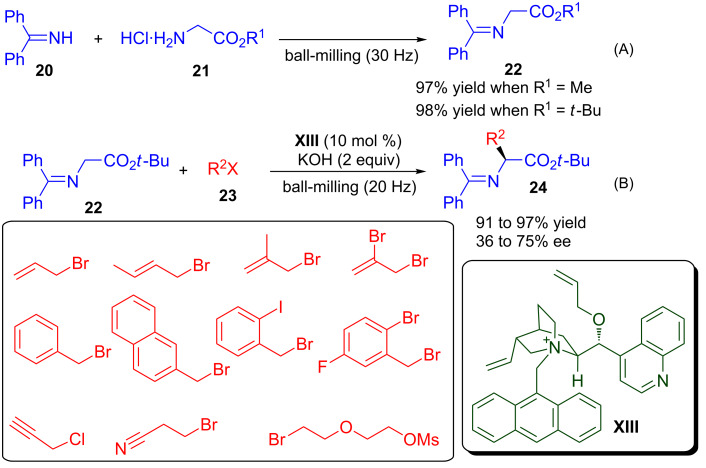
Ball-milling-assisted (A) synthesis of glycine schiff bases and (B) their organocatalytic asymmetric alkylation.

Alkylation of glycine imine by using a chiral phase-transfer catalyst emerged as a very good strategy for the asymmetric synthesis of amino-acid derivatives [59–61]. Lamaty and co-workers prepared a series of glycine Schiff bases **22** in excellent yield (97–98%) in short reaction time by milling protected glycine hydrochloride **21** and diphenylmethanimine (**20**), under solvent-free conditions (Scheme 11) [62]. The asymmetric alkylation of glycine imine **22** was carried out by using a phase-transfer catalyst under basic conditions in a ball-mill. The Schiff base reacted rapidly with various halogenated derivatives **23** in a ball-mill in the presence of KOH and the chiral ammonium salt derived from cinchonidine (**XIII**) as phase-transfer catalyst, to provide excellent yield (91–97%) and good enantioselectivity (35–75% ee) of the corresponding amino esters **24**. Purification of the product was greatly simplified, as equimolar amounts of starting materials were used.

### Asymmetric amination reaction

**Scheme 12:**
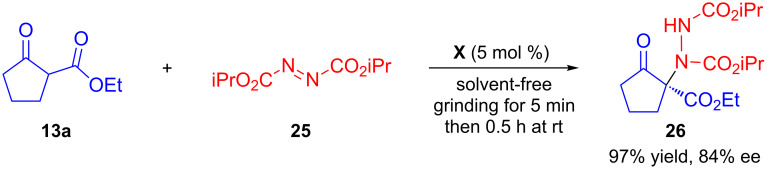
Enantioselective amination of β-ketoester by using pestle and mortar.

Enantioselective organocatalytic amination of trisubstituted β-ketoesters provides an access to quaternary amino stereogenic centres [63–66]. Chimni and Chauhan extended the application of the solvent-free organocatalytic pestle and mortar grinding methodology for the enantioselective amination of β-ketoester **13a** with di-isopropylazodicarboxylate (**25**) (Scheme 12) [48]. Using 5 mol % of **X** the chiral adduct **26** bearing an amino group at a quaternary stereocentre was obtained in 97% yield and 84% ee in short reaction time.

## Conclusion

All the above reports represent a very significant contribution to the development of sustainable asymmetric syntheses. The mechanochemical techniques provide an alternate source of energy, which has been successfully applied in asymmetric organocatalytic reactions. It is very clear that the mechanochemical techniques have an edge over the conventional stirring in terms of higher reaction rate and improved yield. In addition to this, these techniques avoid the use of organic solvents and also facilitate the use of equimolar amounts of reactants, which helps in the easy isolation of products.

Besides the significant progress in the application of mechanochemical techniques in asymmetric organocatalysis, there exists a lot of scope for other asymmetric reactions ranging from simple carbon–carbon, and carbon–heteroatom bond formation to more complex cascade, tandem and multicomponent reactions. However, the stereochemical outcome of many reactions involving chiral organocatalysts is dependent on the reaction conditions and mainly on the nature of the solvents. Thus, it is highly desirable to develop some new organocatalysts whose catalytic efficiency is not dependent on the solvents. Furthermore, the efficiency of some other organocatalytic transformations that were carried out in neat conditions can be increased by performing these reactions with mechanochemical techniques.
